# Does Nursery-Based Intensified Anticipatory Guidance Reduce Emergency Department Use for Nonurgent Conditions in the First Month of Life? A Randomized Controlled Trial

**DOI:** 10.1155/2016/8356582

**Published:** 2016-04-24

**Authors:** Kelly Kamimura-Nishimura, Vikram Chaudhary, Folake Olaosebikan, Maryam Azizi, Sneha Galiveeti, Ayoade Adeniyi, Richard Neugebauer, Stefan H. F. Hagmann

**Affiliations:** ^1^Department of Pediatrics, Bronx-Lebanon Hospital Center, 1650 Selwyn Avenue, Bronx, NY 10457, USA; ^2^College of Physicians and Surgeons, Columbia University, 630 W 168th Street, New York, NY 10032, USA

## Abstract

*Objective.* We aimed to evaluate the impact of an intensified anticipatory guidance program in the nursery on Emergency Department (ED) use for nonurgent conditions (NUCs) in the neonatal period.* Methods*. Parturient mothers of healthy newborns were randomized to an intervention group or control group. Baseline and 1-month follow-up knowledge surveys regarding newborn care were conducted. The primary outcome was the proportion of neonates who used the ED for a NUC. Secondary outcome was change in caregivers' knowledge on NUC.* Results*. Of a total of 594 mothers, 323 (54%) agreed to participate and were randomized to intervention (*n* = 170) or control (*n* = 153) group. Most were Hispanic (68%), single (61%), primiparous (39%), and without high school diploma (44%). 35 (21%) neonates in the intervention group and 41 (27%) in the control group were brought at least once for a NUC to the ED (*p* = 0.12). There was no statistically significant difference in within subject change on knowledge scores between the two study arms.* Conclusions*. Neonatal ED visits for NUCs occur frequently. This nursery-based intensified anticipatory guidance program had no statistically significant impact on neonatal ED use for NUC, nor on neonatal care-relevant knowledge among parturient mothers. Alternative modalities and timing of parental educational intervention may need to be considered. This trial is registered with Clinical Trials Number NCT01859065 (Clinicaltrials.gov).

## 1. Background

Both pediatricians and parents agree that anticipatory guidance is an important component of the well-child visit. However, parents report unmet expectations related to parenting advice, education, or screening during their child's health supervision visit. Few available studies suggest that there may be large variations in the delivery of anticipatory guidance, depending on the population and clinical setting [1, 2]. Paradis et al. found that parents receiving a video intervention rated higher confidence with specific infant care skills and reported feeling better prepared to care for their baby, compared to parents receiving only handouts [3].

First-time parents have many questions about the care of their newborns and most of them are not addressed during the standard follow-up visit at two days or two weeks of age. These concerns bring them into the physician's office or the Emergency Department (ED) for unnecessary visits that could have been resolved at home. Utilization of the ED for nonurgent conditions (NUCs) has long been recognized to be problematic because of cost and lack of continuity of routine care [4]. It has been estimated that about 20 million children in the US are annually brought to the ED for medical care and that up to half of those visits are for NUCs [4]. ED visits for NUCs occur especially frequently in the immediate postnatal period and early infancy [5, 6]. In the literature, management of NUCs in the ED can be considered when a nonurgent triage code was assigned at the time of presentation, no laboratory or radiologic investigations were performed, no physician referral occurred, and the final disposition was to discharge the child home [6, 7].

Newborns obviously represent a unique group of pediatric patients as the increased risk of serious bacterial infection is paired with often only subtle signs of illness naturally leading to heightened parents' anxiety. Indeed, one study showed that more than a third of neonates who returned to the ED within 5 days after being discharged with NUC required subsequent hospitalization [8]. However, there might be an additional burden of newer parents with limited parenting experience or knowledge of their infants' primary pediatric medical home [9].

As this help seeking behavior characterized by unnecessary ED use during the newborn period may be the onset of a pattern of future frequent ED use, we asked whether it could be influenced through a focused educational intervention [7]. Specifically, we aimed to evaluate the impact of an intensified nursery-based anticipatory guidance program on ED use for newborns with NUC.

## 2. Methods

### 2.1. Design

This study was a prospective randomized control trial.

### 2.2. Study Participants

Eligible for participation were English- and Spanish-speaking parturient mothers (aged ≥ 18 years) of healthy full-term newborns (≥36 weeks of gestational age) who received care during the study period at the neonatal service of the Bronx-Lebanon Hospital Center. Inability to converse in either English or Spanish and existing hearing/vision impairments were exclusion criteria.

### 2.3. Recruitment and Randomization

A trained research assistant and the coinvestigators enrolled participants between December 1, 2011, and March 31, 2012. Parturient mothers were approached at the neonatal service on the day of discharge and the ones who agreed to participate in the study were asked to sign an informed consent. The participants were randomly assigned to a control arm receiving a routine anticipatory guidance program or to an intervention arm receiving an intensified anticipatory guidance program. Assignment to respective study arms was determined by month of birth. During months #1 and #3 of the study participant mothers were assigned to the intervention arm, while during months #2 and #4 of the study they were assigned to the control arm. Video-based and handout materials were in English and Spanish.

### 2.4. Interventions

#### 2.4.1. Routine Anticipatory Guidance Control Group

The routine anticipatory guidance program consisted of the already established educational materials (handout and videos) on breastfeeding, reducing the risk of sudden infant death syndrome (from the “Safe to Sleep Campaign”) [10], and prevention of Shaken Baby Syndrome (from the “Portrait of Promise: Preventing Shaken Baby Syndrome”) [11].

#### 2.4.2. Intensified Anticipatory Guidance Intervention Group

The intensified anticipatory guidance program included in addition to the routine anticipatory guidance program a 30-minute video entitled “*Newborn Care: A Guide to the First Six Weeks*” with detailed information based on the latest American Academy of Pediatric safety guidelines on newborns' NUC (e.g., jaundice, well-baby visits, taking temperature, and when to call the doctor) [12]. This video was shown to the participants on the day of discharge by the coinvestigators and/or the research assistant in the nursery.

### 2.5. Outcome Measures

#### 2.5.1. Nonurgent ED Visit

Our primary outcome was to determine the proportion of neonates with at least one nonurgent ED visit during the first month of life. Nonurgent ED use was assessed at 1-month follow-up and by reviewing newborns' electronic medical record. A nonacute ED visit was defined as follows: the infant presented for an acute health concern but a nonurgent triage code at the time of presentation was assigned by the triage nurse, no laboratory or radiologic investigations were performed, and the infant was subsequently discharged home with no physician referral [6, 7].

#### 2.5.2. Maternal Knowledge on Nonurgent Conditions

Knowledge on nonurgent conditions was surveyed using a self-administered, anonymous questionnaire at baseline (before receiving the respective anticipatory guidance program) and at 1-month follow-up (phone interview). After reviewing the 30-minute video, a questionnaire (11 questions) was drawn up to assess maternal knowledge on nonurgent conditions in newborns at baseline and at 1-month follow-up as follows.

1. During feeding, baby should be burped: (a) Never, (b) If breastfed, after changing breast, (c) If formula fed, after 2-3 ounces of formula, (d) (b) and (c), (e) I don't know
2. Where is the best place to take the temperature in a newborn baby? (a) Under arm, (b) Mouth, (c) Buttocks, (d) I don't know
3. A newborn baby has fever when the temperature is above: (a) 98.5 F, (b) 99.4 F, (c) 100.4 F, (d) I don't know
4. This question is to assess your knowledge regarding normal range of stool frequency in newborns: 1 soft stool after 3 days to multiple soft stools in one day: (a) True, (b) False, (c) I don't know
5. This question is to assess your knowledge regarding normal range of wet diapers in newborns: 5–8 wet diapers in one day: (a) True, (b) False, (c) I don't know
6. How should your baby sleep? (a) Sideways, (b) On back, (c) On belly, (c) I don't know
7. Where do you think your baby should be sleeping? (a) Mother's lap, (b) Mother's bed, (c) In his own crib/playpen/bassinet, (d) I don't know
8. When should you call the doctor or take the baby to the Emergency room? (a) Constant/distressed crying, (b) Vomiting/choking, not feeding well, (c) Tightness or shaking or hands or legs, (d) Breathing fast for long time, turning blue, (e) All of the above
9. Which of these is normal for babies? (a) Sneezing, hiccups, (b) Spit up after feeding or during burping, (c) Irregular breathing with no change in skin color, (d) Startle on loud noise or stimulation, (e) All of the above
10. Which of these are normal for a newborn baby? (a) A bluish green or gray birthmark on the lower back or buttocks, (b) A newborn rash or red splotches on skin, (c) Tiny white bumps on the face, (d) Soft spot on the head, (e) All of the above
11. What are the common reasons for crying of normal newborn babies? (a) Wet diaper, (b) Hungry, (c) In pain, (d) Feeling lonely or tired, (e) All of the above.

A composite score (0–11) based on the correct answers on the knowledge questions was calculated. The questionnaire was pretested for clarity and timing in the first 2 weeks of the study period.

### 2.6. Data Analysis

Demographic characteristics were summarized using descriptive statistics. Proportion of newborns who presented for nonurgent ED use at least once during the first month of life in the two trial arms were compared using Fisher's exact test. The difference in within subject nonurgent conditions knowledge score change from baseline to follow-up between the two study groups was assessed using a paired *t*-test. Analyses were based on subjects with complete data on both assessments and on intention to treat with zero score change imputed to subjects absent for the follow-up assessment. We powered our trial for analysis on the primary outcome, nonurgent ED visit. With an anticipated event rate of 20% in the control group, we randomized at least 150 subjects (allowing for a 10% rate of loss to follow-up) to each trial arm to have 95% power with *p* < 0.05, two-sided, to detect a 15% difference between the two arms in the proportions of newborns making at least one nonurgent ED visit [13, 14]. Data were analyzed using SPSS version 19. The hospital's institutional review board approved the study.

## 3. Results

### 3.1. Study Population

**Figure 1:**
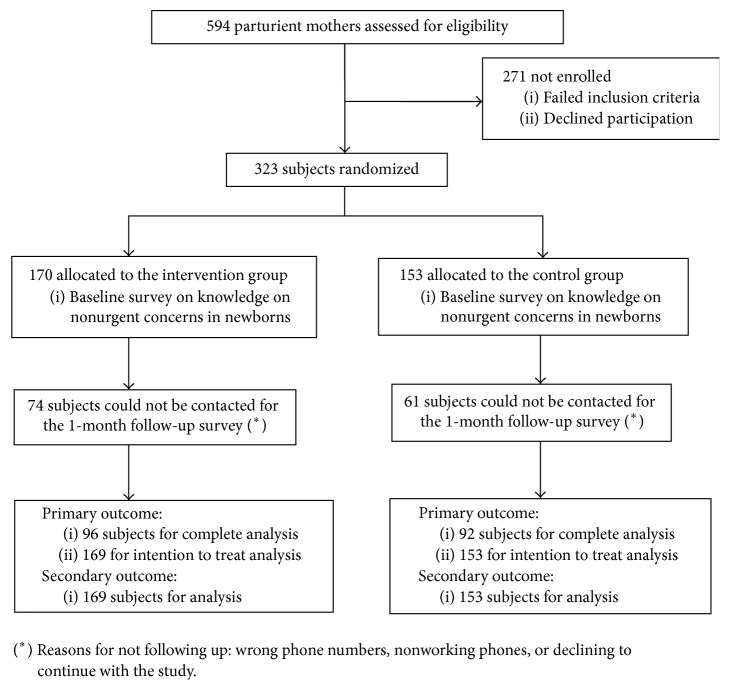
Enrollment flow chart.

Of a total of 594 parturient mothers during the study period, 323 (54%) consented to participate in the study and were randomized to the control (*n* = 153) or the intervention (*n* = 170) group (Figure 1).  Table 1 displays the demographic characteristics of enrolled subjects in each group. The overall mean age was 26.5 years; most were single (61%), of Hispanic ethnicity (68%), and with an incomplete high school education (44%). A third (35%) was new mothers who had just delivered their first child. There were no significant differences between groups regarding age, race/ethnicity, education, marital status, and number of children. 44% (*n* = 74) and 40% (*n* = 61) of the participants were unable to be contacted for the 1-month follow-up survey from the intervention group and the control group, respectively.

### 3.2. Nonurgent ED Visit

Overall, a quarter (76/323, 24%) of the subjects' newborns had at least one nonurgent ED visit reported during the first month of life. While such visits were reported in only 35/170 (21%) infants of the intervention group compared to 41/153 (27%) infants of the control group, this difference was not statistically significant (*p* = 0.12). Subgroup analysis (marital status, level of education, maternal age, ethnicity, and number of children) also did not reveal any statistically significant difference in reported nonurgent ED visits between intervention and control group (data not shown).

### 3.3. Maternal Knowledge on Nonurgent Conditions

There was no significant difference in mean baseline scores between groups (control group: 7.0, SD 2.2; intervention group: 6.9, SD 2.4; *p* = 0.59). Likewise, the mean scores at the 1-month follow-up survey were comparable (control group: 8.5, SD 1.9; intervention group: 8.0, SD 2.2; *p* = 0.11). Further, there was no statistically significant difference in within subject change scores between the two study arms (*p* = 0.52 and *p* = 0.80 for subjects with complete data and intention to treat analysis, resp.).

## 4. Discussion

First and foremost this study documents that about a quarter of newborns in the Bronx are brought to the ED for NUC within the first month of life. Further, we showed that providing parturient mothers in the nursery with intensified anticipatory guidance about such NUC in neonates did not lead to a significant reduction of the rate of nonurgent ED use among the subjects' newborns, nor to superior knowledge gain regarding nonurgent conditions compared to mothers in the control group.

Nationally, ED use overall has continued to rise, with children and especially infants being brought there by caretakers for concern about NUC [15]. A study conducted in Cincinnati, Ohio, more than 10 years ago described that about 20% of infants had an ED visit for a NUC in the first 3 months of life [7]. Our apparent higher rate of this help seeking behavior may be explained by the fact that our institution serves almost exclusively an indigent minority population with poor educational attainment while about half of the subjects in the study from Cincinnati were White [7]. Indeed, non-White race of the mother apart from younger maternal age and Medicaid insurance were identified as significant risk factors associated with ED visits for NUC in the study from Cincinnati [7]. Subgroup analysis in our study failed to find any relevant associations.

With low caregiver health literacy found to be an independent predictor for higher ED use overall and for use of ED for NUC, interventions targeting health literacy skills in parents have been of great interest [16–20]. Preventive pediatric care guidelines by the American Academy of Pediatrics prescribe the discussion of many topics to be covered at each office visit to provide parent with anticipatory guidance [21]. Anticipatory guidance is a developmentally based counseling technique that focuses on the needs of a child at each stage of life [21]. These needs are discussed during well-child care visits to increase parental satisfaction and help them to become a more effective caregiver [1, 2]. Barriers to delivering better anticipatory guidance include limited time and lack of confidence in counseling techniques [22]. However, a recent trial of newborn anticipatory guidance delivered by a DVD during the infant's first visit to the pediatrician's office demonstrated increased parental confidence in specific infant care items that were emphasized in the video and most importantly succeeded in reducing additional health care utilization [13]. We were unable to demonstrate a similar benefit with our video-enhanced anticipatory guidance program on NUC in the nursery. This may be because our study cohort in the South Bronx consisted predominantly of single women who were of Hispanic ethnicity. Both characteristics have been previously identified as leading predictors for seeking care for NUC with children in the ED [23]. Other explanations for lack of impact may be that the added information about NUC was brief, presented only once, and competed with other preventive health messages to be discussed in the nursery (e.g., breastfeeding, SIDS prevention, and Shaken Baby Syndrome) therefore limiting likelihood of parental recall [20, 22]. Indeed, discussing more than 8 anticipatory guidance topics during a pediatric health maintenance visit has not been found to be helpful [2].

Our study has some potential limitations. First, our study may have been underpowered to detect smaller differences in ED use and gained knowledge due to a high attrition rate in both study groups. Second, the intensified anticipatory guidance program that we used in the nursery may not suffice and/or may not be at the right time to improve parental nonurgent conditions knowledge and reduce nonurgent ED visits. Further, during the phone follow-ups, mothers might not remember well or answer truthfully about ED visits of their newborns.

## 5. Conclusions

A significant proportion of healthy newborns in the South Bronx are brought to the ED for a NUC during the first month of life. Incorporating an intensified anticipatory guidance program in the nursery did not have a significant impact on reducing the rate of such ED visits for NUCs nor did it result in an improved gain of NUC-relevant knowledge in parturient mothers. It is possible that the video-enhanced educational program we used in the nursery may not suffice and/or may not be at the right time to improve parental knowledge on NUC and reduce ED use for NUC in our patient population. Introduction of NUC-related topics to future parents using alternative educational modalities (e.g., parenting class) or alternative timing (e.g., pediatric prenatal visit) may be potentially more promising strategies in urban, low-income communities to effect reduced ED use [14].

## Figures and Tables

**Table 1:** Demographic characteristics of all participants in each study group.

Characteristics	All *N* = 323	Control *N* = 153	Intervention *N* = 170	*p* value
Mean age (SD)	26.5 (6.5)	26.6 (6.5)	26.4 (6.5)	0.77

Race/ethnicity (%)				0.16
Hispanic	67.7	64.5	72.0	
African American	25.9	27.6	24.4	
Others	5.6	7.9	5.6	

Education (%)				0.09
Incomplete high school	43.8	37.9	49.1	
Complete high school	22.2	25.5	19.3	
College/postgraduate	34.0	34.5	31.6	

Marital status (%)				0.55
Single	60.8	59.3	62.0	
Married	30.7	30.7	30.7	
Other	8.5	10.0	7.2	

Number of children (%)				0.30
1	34.8	35.1	34.5	
2	30.1	33.1	27.3	
>3	35.1	31.7	38.2	
